# Potential of Cellular Therapy for ALS: Current Strategies and Future Prospects

**DOI:** 10.3389/fcell.2022.851613

**Published:** 2022-03-16

**Authors:** Ting-Jung Lin, Kuang-Chao Cheng, Luo-Yun Wu, Wei-Yu Lai, Thai-Yen Ling, Yung-Che Kuo, Yen-Hua Huang

**Affiliations:** ^1^ School of Medicine, College of Medicine, Taipei Medical University, Taipei, Taiwan; ^2^ Department of Biochemistry and Molecular Cell Biology, School of Medicine, College of Medicine, Taipei Medical University, Taipei, Taiwan; ^3^ TMU Research Center for Cell Therapy and Regeneration Medicine, Taipei Medical University, Taipei, Taiwan; ^4^ Department and Graduate Institute of Pharmacology, College of Medicine, National Taiwan University, Taipei, Taiwan; ^5^ Research Center for Developmental Biology and Regenerative Medicine, National Taiwan University, Taipei, Taiwan; ^6^ International Ph.D. Program for Cell Therapy and Regeneration Medicine, College of Medicine, Taipei Medical University, Taipei, Taiwan; ^7^ Graduate Institute of Medical Sciences, College of Medicine, Taipei Medical University, Taipei, Taiwan; ^8^ Center for Reproductive Medicine, Taipei Medical University Hospital, Taipei Medical University, Taipei, Taiwan; ^9^ TMU Research Center of Cancer Translational Medicine, Taipei Medical University, Taipei, Taiwan; ^10^ Comprehensive Cancer Center of Taipei Medical University, Taipei, Taiwan; ^11^ PhD Program for Translational Medicine, College of Medical Science and Technology, Taipei Medical University, Taipei, Taiwan

**Keywords:** ALS, mesenchymal stem cell, motor neuron degeneration, precision medicine, cellular therapy

## Abstract

Amyotrophic lateral sclerosis (ALS) is a fatal neurodegenerative disease characterized by progressive upper and lower motor neuron (MN) degeneration with unclear pathology. The worldwide prevalence of ALS is approximately 4.42 per 100,000 populations, and death occurs within 3–5 years after diagnosis. However, no effective therapeutic modality for ALS is currently available. In recent years, cellular therapy has shown considerable therapeutic potential because it exerts immunomodulatory effects and protects the MN circuit. However, the safety and efficacy of cellular therapy in ALS are still under debate. In this review, we summarize the current progress in cellular therapy for ALS. The underlying mechanism, current clinical trials, and the pros and cons of cellular therapy using different types of cell are discussed. In addition, clinical studies of mesenchymal stem cells (MSCs) in ALS are highlighted. The summarized findings of this review can facilitate the future clinical application of precision medicine using cellular therapy in ALS.

## Introduction

Amyotrophic lateral sclerosis (ALS) is a fatal neurodegenerative disease characterized by progressive motor neuron (MN) degeneration in the cortex and spinal cord (Brown and Al-Chalabi 2017). Typically, death occurs in 3–5 years after the diagnosis of ALS due to respiratory system dysfunction (Niedermeyer et al., 2019). The overall crude prevalence and incidence of ALS worldwide were 0.442‱ and 0.159‱, respectively (Xu et al., 2020). Compared with women, men are slightly more frequently affected by ALS.

ALS is believed to result from a combination of genetic and environmental factors (Masrori and Van Damme 2020). ALS exists in two forms: familial ALS (fALS) and sporadic ALS (sALS). fALS exhibits a Mendelian pattern of inheritance and accounts for 5–10% of all cases. The remaining 90–95% of cases that do not have an apparent genetic link are classified as sALS (Kiernan et al., 2011). At the genetic level, more than 20 genes have been identified. Among them, chromosome 9 open reading frame 72 (C9ORF72), fused in sarcoma (FUS), TAR DNA binding protein (TARDBP), and superoxide dismutase 1 (SOD1) genes have been identified as the most common causative genes (Riancho et al., 2019). Beyond genetic factors, the diverse pathological mechanisms of ALS-associated neurodegeneration have been discussed (van Es et al., 2017). The clinical symptoms of ALS are heterogeneous, with main symptoms including limb weakness, muscle atrophy, and fasciculations involving both upper and lower MNs. The onset of ALS can be usually divided into two subtypes: spinal and bulbar (Masrori and Van Damme 2020). In addition to MN symptoms, some patients present with frontotemporal dementia, which may cause cognitive and behavioral changes (Grad et al., 2017).

Currently, edaravone and riluzole are the only two drugs approved by the United States Food and Drug Administration (FDA); however, only riluzole can prolong the lifespan of patients for a short time, and edaravone can improve quality of life (Bensimon et al., 1994; Rothstein 2017). In the past few years, increasing attention has been paid to stem cell therapies for ALS because of the differentiation, neuroprotective, and immunomodulatory properties of stem cells (Oskarsson et al., 2018). Several clinical trials have examined multiple types and resources of stem cells, including pluripotent embryonic stem cells (ESCs), induced pluripotent stem cells (iPSCs), multipotent mesenchymal stem cells (MSCs), neural precursor cells, and mononuclear cells (MCs). This review discusses the findings of recent studies on cellular therapies for ALS. Both the potential and challenges are discussed to provide insights into future precision cellular therapeutic strategies.

## Amyotrophic Lateral Sclerosis Pathology

ALS is a complex disease involving multiple genetic risk factors and heterogeneous pathogenic mechanisms. Recent improvements in molecular and genetic technologies have rapidly and substantially advanced the discovery of disease-causing gene mutations and pathways. Understanding disease pathology can maximize the efficacy of treatments and enable the development of innovative therapies for ALS patients.

### The Genetics of Familial Amyotrophic Lateral Sclerosis

Familial ALS (fALS) presents with a family history of the disease, while sporadic ALS (sALS) doesn’t have a hereditary pattern. fALS can be inherited as an autosomal dominant, autosomal recessive, or dominant X-linked type. Adult-onset autosomal dominant inheritance is more common in fALS (Siddique and Ajroud-Driss 2011). Currently, more than 150 disease-causing mutations have been reported. According to a meta-analysis (Zou et al., 2017), mutations in C9ORF72 account for 33.7% of European ALS cases, followed by SOD1 at 14.8%, TARDBP at 4.2%, FUS at 2.8%. Different from their Western counterparts, the Asian population witnessed SOD1 mutations at 30% in familial cases, C9ORF72 at 2.3%, TARDBP at 4.2%, and FUS at 2.8%. On the other hand, mutations in C9ORF72, TARDBP, SOD1, and FUS account for less than 8% of sALS cases in both European and Asian populations.

### Sporadic Amyotrophic Lateral Sclerosis and Pathogenic Mechanisms

The bulk of the genetic determinants of sALS remains elusive, and it is believed that exogenous environmental factors can activate endogenous retroviruses to initiate neurodegenerative changes (Brown and Al-Chalabi 2015). Studies have reported some key features of ALS pathology, such as the involvement of mutated genes encoding RNA-binding proteins including the TARDBP (Sreedharan et al., 2008), fused in sarcoma (FUS) (Vance et al., 2009), and heterogeneous nuclear ribonucleoprotein A1 (HNRNPA1) (Kim et al., 2013). Mutations lead to aberrant RNA metabolism with dysregulated mRNA splicing and transcriptional activity in MNs, thus contributing to protein aggregate formation (Highley et al., 2014; Masuda et al., 2016). A disease hallmark of sALS (up to 97%) cases is TDP-43 proteinopathy (Prasad et al., 2019). Mutations in TDP-43 lead to mislocalization of nuclear TDP-43 to cytoplasmic inclusions and disruption of the autoregulation loop controlling its phase transition (Tziortzouda et al., 2021). The GGGGCC (G_4_C_2_) hexanucleotide repeat expansion in C9ORF72 interacts with RNA-binding proteins and impairs their function (Lee K.-H. et al., 2016). In addition, multiple ALS-causing mutations, such as those in ubiquilin 2 (UBQLN2) (Nishitoh et al., 2008) involved in protein clearance pathways, impair proteostasis and exacerbate protein aggregation. To explain how symptoms spread along neuroanatomical pathways in sALS, it is demonstrated that mutant SOD1 propagate between cells in the motor nervous system in a prion-like manner using SOD1^G85R^ mice (Ayers et al., 2016). Protein aggregates in mitochondria lead to the disruption of the electron transport chain (Choi et al., 2019), resulting in increased reactive oxygen species (ROS) production and mitochondrial dysfunction (Le Gall et al., 2020). Oxidative stress occurs when ROS production exceeds a cell’s capacity to alleviate the stress, thus damaging DNA, proteins, and lipids (Yang 2021). Glutamate excitotoxicity is another main cause of oxidative stress. Because excitatory amino acid transporter-2 functions to uptake glutamate (Lauriat and McInnes 2007), its decreased expression on astrocytes might contribute to high glutamate levels (Rothstein et al., 1995; Gibb et al., 2007), thus resulting in an over influx of Ca^2+^ ions in MNs. The aberrant level of Ca^2+^ further increases ROS production and causes neuronal degeneration (Wang and Qin 2010).

Impaired DNA repair may also contribute to ALS pathogenesis (Mejzini et al., 2019). Because DNA damage and p53 activation mediate the apoptosis of adult MNs (Martin et al., 2005), diseases associated with the nervous system can arise when DNA repair pathways are compromised. Two of the most widely studied ALS-linked proteins, TDP-43 and FUS, prevent or repair DNA damage. TDP-43 is involved in the nonhomologous end joining (NHEJ)–mediated DNA double-strand break repair pathway, whereas FUS interacts with histone deacetylase 1 to regulate DNA damage responses (Mitra et al., 2019). In addition to impaired DNA repair, the G_4_C_2_ repeat expansion in C9ORF72 might alter the intracellular localization of C9ORF72 mRNA, causing nucleocytoplasmic transport defects in ALS (Brown and Al-Chalabi 2017). Although the G_4_C_2_ of C9ORF72 is the most common genetic variant in ALS, the underlying mechanisms remain unclear (Cooper-Knock et al., 2014). RanGAP is a key regulator of nucleocytoplasmic transport. It also suppresses G_4_C_2_-mediated neurodegeneration in *Drosophila* (Cooper-Knock et al., 2014; Zhang et al., 2015). Furthermore, the dysregulation of axonal transport and the axonal compartment are involved in the pathophysiology of ALS (Zhang et al., 2016; Chou et al., 2018). Some pathways may be responsible for impaired axonal transport in patients with mutant SOD1 (Zarei et al., 2015). Studies have revealed that mutant SOD1 inhibits the anterograde axonal transport of mitochondria by reducing the level of mitochondrial Rho GTPase 1 (Miro1) (Moller et al., 2017). Moreover, depletions in two motor molecules, dynein and dynactin-1, can disrupt axonal transport and cause MN degeneration (Ikenaka et al., 2012).

Neuronal injury and death in ALS can be attributed to non–cell-autonomous toxicity, indicating that only gene mutations in motor neurons are insufficient to cause the disease. Activation of glial cells, including microglia and astrocytes, mainly characterizes neuroinflammation in ALS (Vahsen et al., 2021). Two phenotypes of microglia have been reported: M1 and M2; M1 is neurotoxic and produces proinflammatory cytokines and ROS, whereas M2 is neuroprotective and secretes neurotrophic molecules and anti-inflammatory cytokines (Brites and Vaz 2014). The neuroprotective phenotype of microglia is observed in the early stage of ALS, and it switches to the neurotoxic phenotype as the disease progresses, causing neuroinflammation. Recently, this dichotomized classification has been challenged, and the microglia phenotype is considered to be a spectrum in reality (Ransohoff 2016). Similar to microglia, two reactive astrocyte types have been documented: A1 neurotoxic type and A2 neuroprotective type. A2 can promote healing after injury, whereas A1 can be deleterious by reducing the expression of the glutamate transporter GLT-1 (Papadeas et al., 2011), impairing the astrocyte lactate efflux transporter, and activating pro-nerve growth factor-p75 receptor signaling (Ferraiuolo et al., 2011). Recently, the inflammatory niche has been reported to activate microglia to secrete TNFα, IL-1α, and C1q, resulting in the conversion of astrocytes from A2 into A1 (Guttenplan et al., 2020). The neuronal microenvironment is determined by cytokines, which are secreted by surrounding glial cells such as microglia and astrocytes, and it plays a pivotal role in neuroinflammation, neurodegeneration, disease onset, and progression. Current cellular therapies mainly aim to replace damaged MNs or influence the diseased microenvironment through neurotrophic factors produced by differentiated astrocytes and microglia.

From a single initiating event to multiple factors, causative pathogenic mechanisms are yet to have integrated interpretations. Because of genetic and phenotypic variations, additional studies should be performed to elucidate and draw conclusions regarding the pathogenic mechanisms of ALS.

## Therapeutic Strategies

### Symptomatic Care and Pharmacological Strategies

**FIGURE 1:**
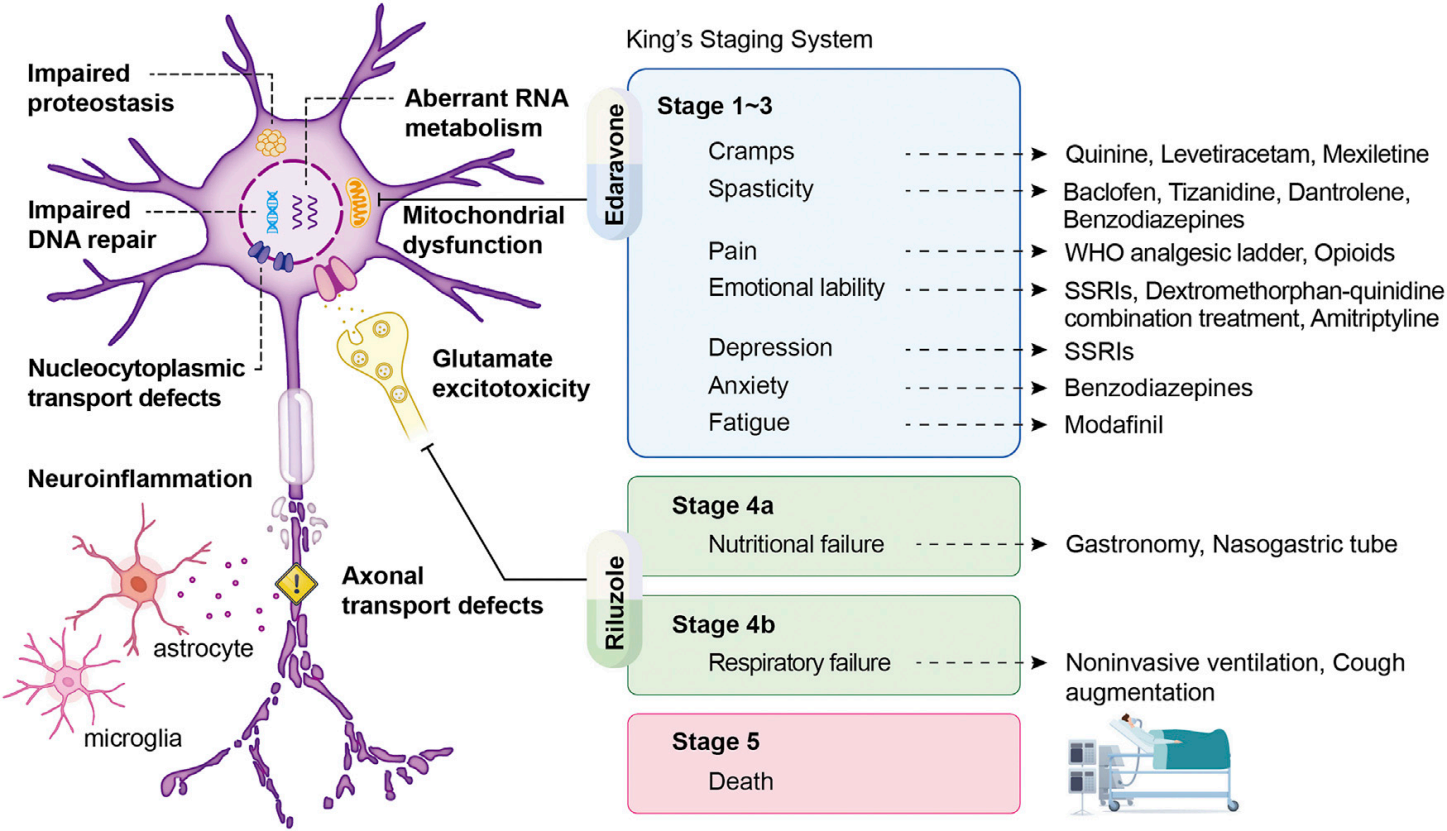
Mechanisms of ALS following treatment to their corresponding symptoms according to King’s staging system. Schematic displaying pathology, symptoms, and therapeutic strategies of ALS, with King’s staging system being presented as the illustration of disease progression. The arrow derives from each symptom points to its therapeutic counterparts. In addition, two FDA-approved drugs, edaravone and riluzole, show considerable benefits in stage 1–3 and stage 4 and are aiming at mitochondrial dysfunction and glutamate excitotoxicity, respectively.

Because no effective cure is available for ALS and some patients have slow disease progression, symptomatic support is crucial for minimizing mortality and improving patients’ quality of life in the disease course (Hobson and McDermott 2016). 75% of patients experience the onset of limb weakness and require the initial focus of care, such as physical therapy and assistive aids, to relieve physical symptoms. Moreover, 25% of patients develop bulbar dysfunction, commonly with dysarthria, dysphagia, weakness, or twitching in the muscles of the tongue (Hobson and McDermott 2016; Institute 2021; Masrori and Van Damme 2020). Therefore, early speech therapy and optimization of communication and nutrition should be provided for these patients (Hobson and McDermott 2016). To alleviate specific symptoms, cannabinoids, tizanidine, baclofen, and muscle stretching can treat spasticity; botulinum toxin injects into the salivary gland or anticholinergic medications can treat sialorrhea. Administration with magnesium supplements, quinine sulfate, gabapentin, or carbamazepine may release muscle cramps. Patients with emotional lability can consider medicating selective serotonin reuptake inhibitors, benzodiazepines, amitriptyline, and dextromethorphan hydrobromide/quinidine sulfate (Masrori and Van Damme 2020). In the past half-century, more than 50 randomized controlled trials of proposed disease-modifying drugs have failed to demonstrate positive results, highlighting our poor knowledge regarding the primary mechanisms of ALS (Petrov et al., 2017; Writing-Group and Edaravone-(MCI-186)-ALS-19-Study-Group 2017). Although numerous candidate drugs for ALS have been identified experimentally, they have failed to show efficacy in human clinical trials, except for riluzole and edaravone (Saitoh and Takahashi 2020) (Figure 1). Riluzole, the first drug for ALS treatment, exhibits neuroprotective activity that is possibly related to the presynaptic inhibition of glutamate damage in the central nervous system. A dose-ranging study reported that treatment with riluzole prolonged stage 4 (King’s clinical staging) not by prolonged stage 2 or 3 or by slowed the disease progression in patients with ALS (Fang et al., 2018). Edaravone (also known as MCI-186), a free radical scavenger, could eliminate peroxynitrite and lipid peroxyl radicals as well as protect MNs from damage caused by free radicals and oxidative stress (Okada et al., 2018; Park et al., 2020; Shefner et al., 2020). However, in the first placebo-controlled phase III study of edaravone, no significant difference in revised ALS Functional Rating Scale (ALSFRS-R) scores was observed between patients receiving edaravone and those receiving placebo. Therefore, whether edaravone is safe and effective in a broader population of patients with advanced ALS stages would require further study (Petrov et al., 2017).

### Gene Therapy

Recently, gene therapy arose as a promising treatment option for ALS patients. Gene therapy can be used to replace the mutated gene with the normal copy, knockout targeting RNA to reduce the expression of causative gene, utilize gene addition to introduce a protective or beneficial factor, or edit the mutant genome (Cappella et al., 2019). Gene therapy delivers genetic material to cells for introducing the functional copies of dysfunctional genes, trophic factors, and other disease-modifying genes, using antisense oligonucleotides (ASOs), RNA interference (RNAi), and gene-editing technology to silence the expression of harmful genes (Amado and Davidson 2021). An ALS-specific challenge must reach both the motor cortex and spinal cord. In order to administrate the material across the blood-brain barrier (BBB) to the motor cortex and spinal cord, intravenous, intracerebroventricular (ICV), intrathecal (IT), intraparenchymal, or intranasal are proved to use.

To target the central nervous system, materials can be delivered naked by using antisense oligonucleotides (ASOs), viral vectors such as an adeno-associated virus, and physical or chemical systems including nanoparticles. Among these, ASOs targeting SOD1 mRNA prolonged survival and improved motor performance in ALS rodent models with SOD1 mutations (Thomsen et al., 2014; McCampbell et al., 2018). Tofersen (BIIB067) is an ASOs targeting SOD1 mRNA by RNase H-dependent degradation (Smith et al., 2006; McCampbell et al., 2018; Rinaldi and Wood 2018) to reduce SOD1 protein concentrations in patients with ALS (Miller et al., 2020). A phase I clinical study revealed that intrathecal delivery of an ASOs targeting SOD1 is safe and no serious adverse events occurred (Miller et al., 2013); another phase I/II clinical study, which is a multiple ascending dose trial, revealed that SOD1 levels are reduced in the cerebrospinal fluid in the patients who received the highest dose of tofersen, and some patients show improvements in clinical function and muscle strength (Miller et al., 2020).

For viral vectors delivery, there is a theoretical advantage that a single treatment can sustain the expression of genetic material in target cells. The adeno-associated viruses have emerged as the leading vectors for CNS delivery due to their ability to transduce terminally differentiated cells and establish nuclear episomes without posing the risk of insertional mutagenesis. Most recently, two patients have intrathecally treated with adeno-associated virus containing an anti-SOD1 microRNA (AAV-miR-SOD1) in an investigator-initiated study. One of the patients shows apparent preservation of strength in his right leg; another patient shows stable scores on a composite measure of ALS function and a stable vital capacity (Mueller et al., 2020). Therefore, the field of gene therapy has the potential for treating MN diseases such as ALS and spinal muscular atrophy. However, the vector should be carefully selected because different viral vectors have pros and cons while delivering the therapeutic gene to the affected region or tissue (O'Connor and Boulis 2012).

## Cellular Therapy

**FIGURE 2:**
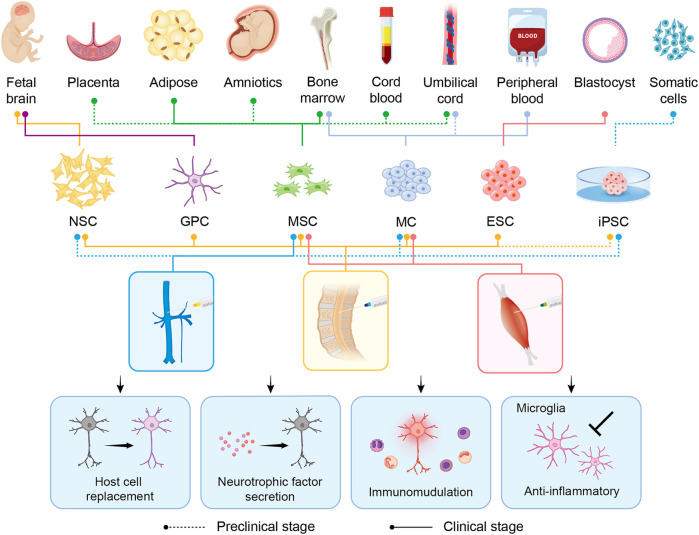
Overview of the derivations, intervention methods, and therapeutic benefits of stem cell transplantation in preclinical/clinical studies related to ALS. Six major candidate cell types (NSC, GPC, MSC, MC, ESC, and iPSC) are represented in the schematic. Different derivations and intervention methods of each cell type are indicated by lines of different colors and types. The type of line, dotted or solid, shows the hierarchy, representing the application in preclinical studies alone or in the clinical stage already. In the middle panel, blue, yellow, and red represent intravenous, intrathecal, and intramuscular injection, respectively. The transplanted cells exert their therapeutic benefits through host cell replacement, neurotrophic factor secretion, immunomodulation, and anti-inflammatory. NSC: neural stem cell; GPC: glial progenitor cell; MSC: mesenchymal stem cell; MC: mononuclear cell; ESC: embryonic stem cell; iPSC: induced-pluripotent stem cell.

Currently, no effective therapeutic options for ALS that can substantially mitigate or even reverse the disease phenotype are available. This may be attributed to our poor knowledge regarding the pathophysiology and variability of ALS (Mitsumoto et al., 2014). Recently, cellular therapy has been the new hope for ALS treatment by targeting multiple pathogenic mechanisms, mainly neuroinflammation (Figure 2). Updated advances, therapeutic potential, and challenges in cellular therapy for ALS are discussed in the following subsections (Tables 1–3).

**TABLE 1:** Preclinical studies of nonhuman cells in rodent models.

Cell Type	Cell Source	Rodent Model	Intervention	Cell Doses (cells)	Results	Ref
ESCs	Mouse ESCs	5–7-week-old Lewis rats	Laminectomy	6 × 10^4^	ESC-derived MNs extend axons into the peripheral nervous system with extensive survival, no neuromuscular junctions were formed	Harper et al. (2004)
ESCs	Mouse ESCs	10-week-old SOD1^G93A^ rats	Laminectomy	1 × 10^5^	Transient improvement of motor function at the early stage	Lopez-Gonzalez et al. (2009)
MSCs	6–8-week-old mouse BMMSCs	90-day-old SOD1^G93A^ mice	Intravenous	1 × 10^6^	Improved survival and motor functions but scantly home to the CNS and poorly engrafted	Uccelli et al. (2012)
MSCs	Rodent BMMSCs	SOD1^G93A^ mice	Intrathecal	1.95 × 10^6^	Decrease motor neuron loss in the lumbar spinal cord, preserving motor functions, and extending the survival of rats	Boucherie et al. (2009)
MSCs	5-week-old rat BMMSCs	SOD1^Leu126delTT^ mice	Intrathecal	4 × 10^5^	Statistically longer disease duration than nontransplanted controls	Morita et al. (2008)
MSCs	16-day-old rat BMMSCs	7-week-old SOD1^G93A^ rats	Intraspinal/intramuscular	1 × 10^5^, 2 × 10^6^	Prolong lifespan and transplanted GFP^+^ MSCs survived in the spinal cord until the end of the disease and migrated both rostrally and caudally from the injection site	Forostyak et al. (2011)
NSCs	6∼8-week-old mouse NSCs	70-day-old SOD1^G93A^ mice	Laminectomy	2 × 10^4^	Delayed disease onset and progression, longer survival, reduced MN loss at the early stage	Corti et al. (2007)
NSCs	Rat NSCs	14/26-week-old SOD1^G93A^ rats	Intravenous	1 × 10^7^	Higher efficiency of cell delivery in symptomatic ALS than presymptomatic ALS, preferentially differentiate into glia	Mitrecic et al. (2010)
NSCs	Mouse NSCs	63-day-old SOD1^G93A^ mice	Intraspinal (Cervical)	2 × 10^5^	Poor cell survival, transient improve of MN function at the early stages of the disease	Srivastava et al. (2017)
GPCs	Rat/mouse GPCs	90-day-old SOD1^G93A^ rats	Intraspinal	9 × 10^5^	Extended survival and disease duration, attenuated MN loss, and slowed declines in forelimb motor and respiratory physiological function	Lepore et al. (2008)
OECs	Mouse OECs	SOD1^Leu126delTT^ mice	Intrathecal	3–4 × 10^5^	No beneficial effect	Morita et al. (2008)
OECs	Rat OECs	100-day-old SOD1^G93A^ rats	Intraspinal	1 × 10^5^	OECs protect neurons, remyelinate axons, adjust microenvironment	Li et al. (2011)
OECs	Rat OECs	90-day-old SOD1^G93A^ rats	Intracranial	5 × 10^5^	Prolonged survival, improved motor skills	Li et al. (2013)
BMMCs	Mouse BMMCs	11-week-old SOD1^G93A^ mice	Intravenous and intramuscular	N/A	Delayed disease progression, decreased microgliosis in the spinal cord, and protection of neuromuscular junction	Gubert et al. (2019)
BMMCs	Mouse BMMCs	9/14-week-old SOD1^G93A^ mice	Intraspinal	1 × 10^6^	Mild transitory delay in disease progression and no increase in lifespan in the presymptomatic phase, and no difference in lifespan or disease progression in the symptomatic phase	Gubert et al. (2016)
BMMCs	Mouse BMMCs	70/110-day-old SOD1^G93A^ mice	Intravenous	1 × 10^7^	Prolonged survival and delayed disease progression in the presymptomatic phase, and a discrete survival increase without other clinical improvements in the symptomatic phase	Venturin et al. (2016)

**TABLE 2:** Preclinical studies of human cells in rodent models.

Cell Type	Cell Source	Rodent Model	Intervention	Cell Doses (cells)	Results	Ref	
iPSCs	hiPSCs	90-day-old SOD1^G93A^ mice	Intraspinal	8 × 10^4^	Differentiation of astrocytes and prolonged lifespan without tumorigenic formation	Kondo et al. (2014)	
iPSCs	hiPSCs	90/105/120-day-old SOD1^G93A^ mice	Intrathecal/intravenous	1 × 10^6^	Improved neuromuscular function and survival, neuroprotection, and positive host-environment modifications	(Nizzardo et al., 2014)	
iPSCs	hiPSCs	90-day-old SOD1^G93A^ mice	Intrathecal	1 × 10^6^	Increased survival and improved neuromuscular phenotype	Nizzardo et al. (2016)	
iPSCs	hiPSCs	3-month-old SOD1^G93A^ rats	Intraspinal	1 × 10^5^	Successful survival and differentiation into mature neurons	Popescu et al. (2013)	
MSCs	hUCMSCs	98-day-old SOD1^G93A^ mice	Multiple intracerebroventricular injections	2.5 × 10^5^	No transplanted cells migrate to the spinal cord, a partial but significant protection of MNs was detected (anti-inflammatory, neuroprotective) in the lumbar spinal cord. Did not prevent muscle denervation nor delayed disease progression	Sironi et al. (2017)	
MSCs	hADMSCs	8-week-old SOD1^G93A^ mice	Intraperitoneally	1 × 10^6^	No significant difference was observed in the survival of mice treated with MSCs	Coatti et al. (2017)	
MSCs	hBMMSCs	SOD1^G93A^ rats	Intrathecal	5 × 10^5^	Ameliorated disease progression, significantly improved motor activity, prolonged survival	Forostyak et al. (2014)	
MSCs	hBMMSCs	SOD1^G93A^ mice	Intravenous	1 × 10^6^	MSCs-Ngn1 delayed disease onset, enhanced motor functions and can migrate to the CNS	Chan-Il et al. (2013)	
MSCs	hBMMSCs-GDNF	SOD1^G93A^ rats	Intramuscular	1.2 × 10^5^	Ameliorate motor neuron loss, delayed disease progression, increased overall lifespan by up to 28 days	Suzuki et al. (2008)	
MSCs	hBMMSCs	mdf/ocd mutant mice	Intrathecal	5 × 10^5^	Ameliorating the symptoms of a motor neuron degenerative mouse model and a less degree of MSCs improved significantly in the motor tests performed	Boucherie et al. (2009)	
MSCs	hBMMSCs	2-month-old SOD1^G93A^ mice	Cisterna lumbaris injection	3 × 10^5^	Motoneuron death and motor decay were delayed, astrogliosis was reduced, and microglial activation was modulated	Boido et al. (2014)	
NSCs	hNSCs	62-day-old SOD1^G93A^ rats	Lumbar puncture	4 × 10^5^	Prolonged lifespan, delayed motor neuron death, disease onset, and progression	Xu et al. (2006)	
NSCs	hNSCs	56-day-old SOD1^G93A^ rats	Lumbar puncture	1.6 × 10^5^	Advanced degree of structural integration between grafted cells and host ones, prolonged lifespan, delayed motor neuron death, disease onset, and progression	Xu et al. (2009)	
NSCs	hNSCs-VEGF	70-day-old SOD1^G93A^ mice	Intrathecal	1 × 10^5^	Delayed disease onset, prolonged survival, provided neuroprotective effect	Hwang et al. (2009)	
NSCs	hNSCs	63-day-old SOD1^G93A^ rats	Laminectomy	2.4 × 10^5^	Prolonged lifespan, extended disease duration, delayed disease onset, attenuated motor weakness	Xu et al. (2011)	
NSCs	hNSCs	60–65-day-old SOD1^G93A^ rats	Laminectomy	1 × 10^5^	Transient local improvement of MNs, no survival benefit, no delayed onset/progression	Hefferan et al. (2012)	
NSCs	hNSCs	8-week-old SOD1^G93A^ rats	Lumbar puncture	8 × 10^4^	Stimulate endogenous neurogenesis, initiate intrinsic repair mechanisms	Xu et al. (2012)	
GPCs	hGPCs	70-day-old SOD1^G93A^ rats	N/A	1.456 × 10^6^	Increased motor neuron survival, no effect on the loss of muscle innervation	Suzuki et al. (2007)	
GPCs	hGPCs	75-day-old SOD1^G93A^ mice	N/A	N/A	Attenuated the loss of MNs, induced trophic changes in MNs, no improvement in motor performance and extension of lifespan	Park et al. (2009)	
GPCs	hGPCs	50–60-day-old SOD1^G93A^ mice	Laminectomy	2 × 10^5^ 6 × 10^5^	No motor neuron protection or any therapeutic benefits	Lepore et al. (2011)	
GPCs	hGPCs	80-day-old SOD1^G93A^ rats	Laminectomy	4 × 10^5^	Delayed disease onset, extended survival, improved health of MNs	Thomsen et al. (2018)	
UCBCs	hUCBCs	5∼6-week-old SOD1^G93A^ mice	N/A	1 × 10^8^	Improved probability of neuromuscular transmission	Souayah et al. (2012)	
UCBCs	hUCBCs	22–25-week-old SOD1^G93A^ mice	N/A	1 × 10^6^	UCBCs transfected with VEGF and ${\text{L}}_{1}$ CAM differentiated into endothelial cells, formed new blood vessels and secreted neuro-trophic factors, supporting neurogenesis	Rizvanov et al. (2008)	
UCBCs	hUCBCs	8-week-old SOD1^G93A^ mice	N/A	3.42–3.56 × 10^6^	Considerably delayed onset of symptoms and death	Ende et al. (2000)	
UCBCs	hUCBCs	7∼8-week-old SOD1^G93A^ mice	Intravenous	1 × 10^7^ 2.5 × 10^7^ 5 × 10^7^	Increased lifespan, delayed disease progression, decreased proinflammatory cytokines in the brain and spinal cord, higher response of splenic cells to mitogens, significantly increased lymphocytes and decreased neutrophils in the peripheral blood, stable reduction in microglia density in both cervical and lumbar spinal cords	Garbuzova-Davis et al. (2008)	
UCBCs	hUCBCs	45-day-old SOD1^G93A^ mice	Intrathecal	1 × 10^5^	Narrowed therapeutic effects due to limited intraparenchymal migration and survival	Habisch et al. (2007)	
UCBCs	hUCBCs	9/13-week-old SOD1^G93A^ mice	Intravenous	1 × 10^6^ 2.5 × 10^6^	Delayed functional deterioration, increased lifespan, higher motor neuron counts, reduced astrocytes and microglia	Garbuzova-Davis et al. (2012)	
UCBCs	hUCBCs	29-week-old SOD1^G93A^ mice	Intravenous	2 × 10^6^	Increased life span and performance in behavioral tests	Islamov et al. (2017)	

**TABLE 3:** Clinical trials of cellular therapy in ALS patients.

Cell Type	Cell Source	Phase	Intervention	Cell Doses	Recruit	Location	Results	NCT Number
ESCs	Nonautologous ESCs	1/2	Intrathecal	N/A	16	Israel	N/A	03482050
MSCs	Autologous BMMSCs	3	Intrathecal	N/A	261	United States	N/A	03280056
MSCs	Autologous BMMSCs	1/2	Intrathecal	1 × 10^6^ cells/kg	20	United States	N/A	04821479
MSCs	Autologous MSCs	1	Intrathecal	1 × 10^8^ cells/kg	3	Brazil	N/A	02987413
MSCs	Autologous MSC-NTF	2	Intrathecal/intramuscular	N/A	48	United States	N/A	02017912
MSCs	Autologous BMMSCs	1	Intrathecal	N/A	8	Iran	N/A	01771640
MSCs	Autologous BMMSCs	1	Intrathecal	1 × 10^6^ cells/kg	1	United States	N/A	01142856
MSCs	Autologous ADMSCs	1	Intrathecal	1 ×10^7^ cells/kg; 5 × 10^7^ cells/kg; 1 × 10^8^ cells/kg	27	United States	N/A	01609283
MSCs	Nonautologous ADMSCs	1	Intravenous	2 × 10^6^ cells/kg	19	Iran	N/A	02492516
MSCs	UCMSCs	2	Intrathecal	N/A	30	China	N/A	01494480
MSCs	Autologous BMMSCs	1	Intrathecal	N/A	30	Poland	N/A	02881489
MSCs	BMMSCs	1/2	Intrathecal	N/A	28	Brazil	N/A	02917681
MSCs	Autologous ADMSCs	2	Intrathecal	1–10 × 10^7^ cells	60	United States	N/A	03268603
MSCs	Wharton’s Jelly-derived MSCs	1/2	Intrathecal	N/A	20	Poland	N/A	04651855
MSCs	Nonautologous UCMSCs	1	Intrathecal	5 × 10^7^ cells	20	Antigua and Barbuda	N/A	05003921
MSCs	Wharton’s Jelly-derived MSCs	1	Intrathecal	N/A	30	Poland	N/A	02881476
MSCs	Autologous BMMSCs	3	Intrathecal	N/A	115	Korea	N/A	04745299
MSCs	Autologous BMMSCs	1/2	Intrathecal	15 ± 4.5 × 10^6^ cells	26	Prague	No severe adverse reaction, slow down the progression of the disease	03828123
MSCs	Autologous BMMSCs	1/2	Intramuscular	1 × 10^6^ cells/kg	12	Israel	Safe, 25% or more reduction in ALS-FRS-R slope	01051882
NSCs	Nonautologous NSCs	1	Intraspinal	5–15 × 10^5^ cells	15	United States	No acceleration of disease progression, improved survival and function compared with historical datasets	01348451
NSCs	Nonautologous NSCs	1	Intraspinal	2.25 × 10^6^ cells 4.5 × 10^6^ cells	6	Italy	No acceleration of disease progression, no tumor formation, slight improvement in three patients	01640067
NSCs	Nonautologous NSCs	2	Intraspinal	2 × 10^6^ cells; 4 × 10^6^ cells; 6 × 10^6^ cells; 8 × 10^6^ cells; 16 × 10^6^ cells	15	United States	Well-tolerated high cell doses, safe expansion to multiple centers, improved survival and function compared with historical datasets	01730716
GPCs	Nonautologous GPCs	1/2a	Intraspinal	N/A	18	United States	N/A	02943850
GPCs	Nonautologous GPCs	1/2a	Intraspinal	N/A	30	United States	N/A	02478450
BMMCs	Autologous BMMCs	1/2	Intramuscular	5.5 × 10^8^ cells	22	Spain	No effect on the tibialis anterior muscle motor unit properties, higher 50 index	02286011
BMMCs	Autologous BMMCs	1	Intraspinal	4.62 × 10^8^ cells	11	Spain	Reduction of TDP-43 deposits in the spinal motoneurons	00855400
BMMCs	Autologous BMMCs	2	Intramuscular	N/A	100	Spain	Safe, and a positive transitory effect on the size and number of motor units of the TA muscle	04849065
BMMCs	Autologous BMMCs	1/2	Intrathecal	N/A	63	Spain	N/A	01254539
PBMCs	Autologous PBMCs	N/A	Intraspinal	1 × 10^9^ cells	14	China	Safe, but therapeutic effect is not remarkable	03085706

### Embryonic Stem Cells

ESCs are derived from the inner cell mass of a developing blastocyst and possess the ability of self-renewal and pluripotency; in other words, they can develop into any cell type in the human body indefinitely (Young 2011). However, such a powerful capability is a double-edged sword because plasticity not only permits ESCs to be employed in several scientific domains such as human developmental biology, drug discovery, and transplantation therapy but also makes them difficult to control (Thomson et al., 1998; Murry and Keller 2008). Over the last 3 decades, scientists have been decoding the developmental principles of ESCs, in order to convert them into functionally specialized cells. The translation of tuning cell fate from prospect to reality was eventually achieved, thus indicating that ESCs could be artificially induced to differentiate into the ectoderm and further generate specific neurons and glial cells (Wichterle et al., 2002; Jiang et al., 2012). Hence, ESCs have been widely utilized to investigate the mechanisms of ALS by modeling the disease *in vitro* (Clement et al., 2003; Marchetto et al., 2008). Moreover, ESCs can be used in potential therapeutic molecule screening as an approach for testing its efficacy (Yang et al., 2013). Targeting the replacement of MNs or the neuroprotective functions of glial cells, ESCs are considered to be a potential stem cell resource for ALS treatment.

Studies have reported the possible therapeutic benefits of ESCs. A preclinical study indicated that ESC-derived MNs could be induced to extend axons in the peripheral nervous system but without neuromuscular junctions (Harper et al., 2004). Furthermore, López-González et al. determined that engrafted ESC–derived MNs in SOD1^G93A^ rodent models resulted in the transient recovery of motor function in the early stage of ALS. The potential mechanism involves the trophic support provided by transplanted cells for deteriorating endogenous MNs (Lopez-Gonzalez et al., 2009). In addition to ESC-derived MNs, ESC-derived astrocytes were studied. A phase I/IIa trial recruited 16 patients with ALS to receive human ESC-derived astrocyte treatment (NCT03482050, 2018), aiming for the functional compensation of host astrocytes.

Although ESCs can be useful in cellular therapy, ethical issues and safety concerns have been reported. Ethical problems arise from the destruction of embryos, thus leading to the debate of when human life begins (Lo and Parham 2009). In terms of safety, the tumorigenicity of ESCs is the main issue. Because ESCs share cellular and molecular phenotypes with tumor cell lines, they form teratomas after transplantation (Ben-David and Benvenisty 2011). These uncontrollable uncertainties harbor risks, and ethical controversies must be appropriately addressed. ESCs cannot be used in clinical practice until the aforementioned difficulties are overcome in the future.

### Induced Pluripotent Stem Cells

Induced Pluripotent Stem Cells have considerably altered the landscape of stem cell research (Malik and Rao 2013). They present ESC-like properties, including unlimited expansion and the capacity to differentiate into three germ layers (Takahashi and Yamanaka 2006). Compared to ESCs, the circumvention of ethical dilemmas and graft rejection strongly drives the potential of iPSC therapy to be a promising approach (Goutman et al., 2019). ALS-related iPSCs were first generated using skin biopsies and were successfully differentiated into MNs (Dimos et al., 2008). In addition, iPSCs from patients with mutated SOD1 (Chen et al., 2014), C9ORF72 (Donnelly et al., 2013), TARDBP (Bilican et al., 2012), FUS (Guo et al., 2017), VABP (Mitne-Neto et al., 2011), and sporadic ALS (Fujimori et al., 2018) have been reported. iPSC-derived MNs or astrocytes present the disease pathology and can thus be used as a disease cell model for studying the pathogenic mechanisms.

The combination of iPSCs and CRISPR/Cas9 facilitates the investigation of disease-causing mutations (Yun and Ha 2020). Because of the high efficiency of disease modeling, iPSC-based drug screening is well developed (Egawa et al., 2012). Among the pharmacological candidates, ropinirole (Fujimori et al., 2018), bosutinib (Imamura et al., 2017), and ezogabine (USAN)/retigabine (INN) (Wainger et al., 2014) are considered potentially beneficial for ALS patients. Compounds such as HDAC6 inhibitors (Guo et al., 2017) and apilimod (Shi et al., 2018) have also been screened by the iPSC platform. In addition, iPSCs have considerable potential for use in the autologous cellular transplantation strategy for ALS. Their ability to differentiate into neural precursor cells provides therapeutic benefits, including the replacement of endogenous cells and the modulation of the host environment in neurodegenerative diseases (Xiao et al., 2015).

Several preclinical studies using skin fibroblast-derived iPSCs have reported the therapeutic potential of iPSCs. A study transplanted human iPSC (hiPSC)-derived neural stem cells (NSCs) into the spinal cord and found that hiPSC-NSCs could survive and differentiate into neurons in the ALS microenvironment (Popescu et al., 2013). In addition, both IT and intravenous injections of hiPSC-NSCs could ameliorate the ALS disease phenotype and increase the lifespan of rodent models, with no side effects (Nizzardo et al., 2014). Novel axonal and collateral sprouting could be induced after engraftment (Nizzardo et al., 2016). Besides, hiPSC-derived glial progenitor cells (GPCs) could induce astrocyte proliferation, prolong the lifespan, and improve lower limb function transiently after being transplanted into the lumbar spinal cord of SOD1^G93A^ mice. Furthermore, AKT-VEGF signaling may be involved in the underlying mechanism of therapeutic effects (Kondo et al., 2014).

Although some preclinical studies have examined the efficacy of iPSCs for ALS treatment, no clinical trial is yet approved. Studies have described some concerns. The first concern is the tumorigenicity of iPSCs. Three tumorigenic scenarios are observed: the formation of teratomas, the active state of reprogramming factors, and genetic abnormalities during *in vitro* culture. In addition, the heterogeneity of iPSCs contributes to defects in the differentiation of some cell lines, thus resulting in the formation of teratomas (Yamanaka 2020). Second, deriving autologous iPSCs from each individual is expensive and requires a long cell cultivation period. Moreover, allograft approaches may cause immune rejection (Takahashi and Yamanaka 2013; Yamanaka 2020). Despite the existing challenges, iPSC technology holds great potential in many domains related to ALS cellular therapy. For instance, iPSC models can enhance the investigation of disease progression, thus enabling the early diagnosis of ALS; personalized medical strategies for patients with sporadic ALS might be developed through high-throughput screening (Coatti et al., 2015). Although more studies should be performed to determine the feasibility of iPSC engraftment, the application of iPSCs already offers a new path for personalizing ALS cellular therapy.

### Mesenchymal Stem Cells

Nowadays, MSCs are considered a promising tool for ALS cellular therapy due to the following reasons: 1) MSCs can be effectively and safely obtained from somatic tissues and can be easily expanded *in vitro* culture systems; 2) MSCs can migrate to injury tissue sites, a property called “homing” that is mediated by chemokines; 3) MSCs can release various biological factors to facilitate nervous tissue maintenance and repair; 4) MSCs may develop neuronal phenotypes under appropriate conditions; 5) MSCs have lower ethical concerns than ESCs or fetal-derived stem cells; 6) autologous MSCs can be safely administered without the risk of immune rejection; and 7) MSCs can secrete exosomes that modulate the microenvironment (Karussis et al., 2010; Baglio et al., 2012; Zhou et al., 2021).

Mesenchymal stem cells can be isolated from different somatic tissues including the peripheral blood, cord blood, umbilical cord (Wharton’s jelly), placenta, amniotic fluid, fetal tissues, adipose tissue, dental pulp, skeletal muscle, periosteum, lung, synovial fluid, and cartilage (Wang et al., 2004; Kusuma et al., 2017; Zhuang et al., 2021). Unlike other stem cells, MSC targets could change the cellular environment of vulnerable brain areas when used for treating neurodegenerative diseases in preclinical trials (Chen et al., 2018). The potential therapeutic effects of MSCs on ALS may involve rich trophic factor secretion; enhanced neurogenesis; abnormal protein aggregate clearance; immunomodulation by the increased expression of interleukin (IL)-10 and IL-6, vascular endothelial growth factor (VEGF), and transforming growth factor beta-1 (TGF-β1); and gene delivery or replacement of lost cells (Lewis and Suzuki 2014; Nabavi et al., 2019). MSCs exert neuroprotective effects on glutamate excitotoxicity by inhibiting the expression of the N-methyl-D-aspartate receptor (NMDAR) and by controlling glutamate-related Ca^2+^ influx (Voulgari-Kokota et al., 2012). Thus, the therapeutic plasticity of MSCs matches the complexity of ALS, making MSCs a strong candidate for ALS treatment.

Mesenchymal stem cells regulate both adaptive and innate immune reactions through paracrine such as TGF-β secretion. An increase in regulatory T cells and Th2 cells play crucial roles in the neuroprotective effect on motor neuronal cell death mechanisms in ALS (Kim S. H. et al., 2018). MSCs can suppress T-cell function, dendritic cell maturation, B-cell proliferation, and natural killer cell cytotoxicity; promote regulatory T cell proliferation; and trigger M1 to M2 macrophage conversion (Shariati et al., 2020). The repeated infusion of MSCs could reduce the deterioration of ALS motor function and prolong life expectancy, whereas a single infusion of MSCs could delay disease progression (Magota et al., 2021a; Magota et al., 2021b). Overexpression of the hepatocyte growth factor, glial cell line-derived neurotrophic factor (GDNF), and insulin-like growth factor in MSCs could substantially reduce the progression of motor dysfunction (Terashima et al., 2020). Intramuscular transplantation of human umbilical cord (hUC)-derived MSCs could ameliorate muscle atrophy and reduce neuromuscular degeneration in the skeletal muscle by reducing intracellular ROS levels (Kook et al., 2020). Repeated ICV injections of hUC-derived MSCs in SOD1G93A mice did not result in the migration of transplanted cells to the spinal cord or delay muscle denervation but provided protection the lumbar spinal cord with anti-inflammatory (IL-4 and IL-10) and neuroprotective (IGF-1) microenvironment instead of the pro-inflammatory (IL-6 and IL-1β) stimuli (Sironi et al., 2017). In patients with ALS, 50% of pericytes, which are responsible for the formation and maintenance of the BBB, from the spinal cord barrier are lost (Coatti et al., 2017). Disruption of the BBB including decreases in the levels of tight junction proteins, immunoglobulin G, and hemosiderin perivascular deposits and the expression of GLUT1 precedes neuroinflammation, MN loss, and motor impairment (Sweeney et al., 2019). In a preclinical study, both adipose-derived pericytes and MSCs were intraperitoneally administered to SOD1G93A mice. The results showed that pericytes significantly prolonged survival in mice, whereas MSCs showed no effect on survival (Coatti et al., 2017). In addition to the loss of the BBB, changes in the ventral horn perineuronal net structure were observed in SOD1 rats; this structure has been recently shown to protect against various toxic insults, and it is affected in neurodegenerative diseases (Cabungcal et al., 2013; Suttkus et al., 2016). Forostyak et al. (2014) demonstrated that the IT delivery of MSCs in SOD1^G93A^ rats preserved the perineuronal net structure and altered cytokine homeostasis accompanied by the prolonged survival of MNs (Forostyak et al., 2014).

Aside from cell transplantation, one of the reasons why MSCs have been highlighted in recent years is their high capacity for secreting exosomes. Exosomes are a kind of extracellular vesicles that are responsible for intercellular communication by carrying numerous molecules, including lipids, DNA, mRNA, miRNA, receptors, growth factors, immunomodulators, and antioxidants, which perform a variety of functions such as neuroprotection and anti-inflammation (Andjus et al., 2020; Sykova et al., 2021). Because of the exosome small size, it can cross the BBB to modulate the disease microenvironment as cell-free therapeutic tools (Bonafede and Mariotti 2017). Previous *in vitro* studies demonstrated that exosomes produced by adipose-derived MSCs could protect MN-like cells with ALS mutations from oxidative damage (Bonafede et al., 2016), play anti-apoptotic effect (Bonafede et al., 2019), and alleviate SOD1 aggregation and mitochondrial dysfunction (Lee M. et al., 2016). An *in vivo* study indicated that repeated administration of exosomes from adipose-derived MSCs protects MNs and home to damaged regions in SOD1^G93A^ models (Bonafede et al., 2020). Therefore, exosomes secreted by MSCs might be a novel therapeutic candidate for ALS.

Many clinical studies have examined the safety and efficacy of MSCs. In phase I clinical study, safety evaluations reveal that there are no severe transplant-related adverse events and no structural changes in the brain or the spinal cord such as tumor formation. (Mazzini et al., 2010). Repetitive injections of cells have commonly been used in cellular therapy; for example, repeated injections of autologous bone marrow-derived MSCs (BM-MSCs) are showing no significant adverse events (Nabavi et al., 2019; Siwek et al., 2020). A phase II clinical trial with repeated intrathecal administration of BM-MSCs in patients with ALS is safe and well-tolerated (Petrou et al., 2021). Intriguingly, a case report has shown that intraventricular transplantation of autologous BM-MSCs using the Ommaya reservoir in a patient with ALS is safe, and this device has the potential to make repetitive injections of cells more easily (Baek et al., 2012). Moreover, MSC-neurotrophic factor (NTF) cells (NurOwn^®^, autologous BM-MSCs) have been induced to secrete high levels of NTFs. NTFs are small, naturally occurring polypeptides that support the development and survival of neurons (Henderson 1996). MSC-NTFs could offer a novel method for simultaneously delivering multiple NTFs to patients with neurodegenerative diseases such as ALS while playing the potential immunomodulatory therapeutic benefits of MSCs (Gothelf et al., 2017). MSC-NTFs cells (NurOwn^®^) have been successfully used in a phase Ⅱ clinical trial (NCT02017912) and proved its safety and early promising of efficacy in patients with ALS (Berry et al., 2019). Most recently, a randomized phase III controlled clinical trial comparing NurOwn^®^ to placebo has also been completed (NCT03280056) in patients with ALS. The results do not reach statistical significance on the primary endpoint; nevertheless, patients who received NurOwn^®^ have been significantly improved in cerebrospinal biomarkers of neuroinflammation, neurodegeneration, and neurotrophic factor support (Cudkowicz et al., 2021). Although this trial does not support the proposed clinical benefit of cellular therapy for ALS, NurOwn^®^ is warranted for further investigation.

Reducing the expression levels of pro-inflammatory cytokines and increasing anti-inflammatory cytokines are observed after administration of MSCs (Oh et al., 2018). MSCs could stimulate intrinsic neurogenesis, and they released diverse neurotrophic factors and modulated immunoinflammatory processes (Uccelli et al., 2011; Keating 2012). Previously, 12 patients were enrolled to analyze their lymphocyte subsets and cytokine production after MSCs administration and demonstrated that MSCs caused immediate *in vivo* immunomodulation. This effect of MSCs was observed as early as 4 h after MSC transplantation. For example, the proportions of CD4^+^/CD25^+^ regulatory cells and activated dendritic cells are increased and decreased, respectively. Moreover, lymphocytes and lymphocyte proliferation are also reduced at 4 h after MSC transplantation (Karussis et al., 2010). Previous, a clinical trial has combined two cellular therapy protocols: T-cell vaccination (TCV) and BEN (BM-MSCs, effector T cells, and neuroblasts) approach in patients with ALS, and found that the combinational treatment is safe, feasible, and effective. This therapy might restore some dead MNs with clinical significance (Moviglia et al., 2012).

Many limitations still exist in the clinical application of MSCs. Studies examining the effects of MSCs on ALS applied different clinical parameters, and the higher efficacy of MSCs can be ensured by optimizing the treatment timing, MSC types, and treatment route and by examining sex differences in further translational studies. The preparation of BM-MSCs for patients with rapid disease progression is challenging and time-consuming. Thus, other allogeneic sources of MSCs should be identified for this type of therapy (Siwek et al., 2020). Moreover, immunomodulatory effects of ALS patient-derived MSCs are reduced due to their higher levels of pro-inflammatory cytokines. Nevertheless, the therapy effectiveness of autologous MSCs of ALS patients under the pro-inflammatory microenvironment deserves more studies and investigations (Javorkova et al., 2019).

### Mononuclear Cells

Mononuclear cells (MCs) can be isolated from the peripheral blood, human umbilical cord blood, and bone marrow in the following paragraphs, and they are abbreviated as PBMCs, hUCBMCs, and BMMCs, respectively. Rather than a specific cell type, mononuclear cells are a heterogeneous population composed of hematopoietic stem cells (HSCs), hematopoietic progenitors, a small number of MSCs, and differentiated bone marrow cells including monocytes and lymphocytes (Vasques et al., 2021). With its diverse composition, mononuclear cells are useful considering the multi-etiological and non-cell-autonomous nature of ALS.

Many ALS preclinical trials have tested different mononuclear cell sources, cell doses, and administration routes. First, high doses of irradiation were used in SOD1^G93A^ mouse models to replace the bone marrow with BMMCs (Corti et al., 2004). The results revealed an increase in animal survival. Two studies have suggested that BMMC treatment delayed disease progression, with more favorable outcomes noted in the presymptomatic phase than in the symptomatic phase (Gubert et al., 2016). Administering BMMC therapy through different routes (intravenous and intramuscular) simultaneously not only delayed disease progression but also reduced microgliosis in the spinal cord and protected the neuromuscular junction (Gubert et al., 2019). A phase II clinical study that injected bone marrow-derived HSCs into the anterior part of the spinal cord of 13 patients with ALS. Nine of 13 patients show much better outcomes, indicating that this strategy is safe and effective (Deda et al., 2009). Moreover, in a pilot safety study, Blanquer et al. (NCT00855400) showed a reduction in TDP-43 deposits in the spinal motoneurons of patients (Blanquer et al., 2012). A retrospective controlled study indicated that patient survival was significantly extended by BMMC therapy (Sharma et al., 2015). The safety and feasibility of BMMCs are thereby confirmed, and further research must be conducted to continually investigate the neuroprotective mechanism as well as to determine the best protocol for clinical practice.

Next, in a preclinical trial, Ende et al. reported that the use of hUCBMCs in SOD1^G93A^ mouse models considerably delayed the onset of ALS symptoms and death (Ende et al., 2000). The optimal dose of hUCBMCs was found to be 2.5 × $10^{7}$ cells, which obviously prolonged the lifespan of mice by 20–25%, delayed disease progression by 15%, and decreased proinflammatory cytokines in the brain and spinal cord (Garbuzova-Davis et al., 2008). hUCBMCs is considered preferable to other cell sources because they are not only low in terms of pathogenicity but also express lower levels of cytokine receptors (IL-2, IL-4, IL-6, IL-7, TNF, and IFNγ) and higher levels of anti-inflammatory cytokines than adult blood cells (Zola et al., 1995). In addition, Islamov et al. co-transduced hUCBMCs with adenoviral vectors expressing GDNF, VEGF, and neural cell adhesion molecule and observed a prominent increase in ALS mice lifespan after presymptomatic treatment (Islamov et al., 2017). Rizvanov et al. discovered that hUCBMCs transfected with VEGF and ${\text{L}}_{1}$ CAM produced neuroprotective effects in mouse models because hUCBMCs could differentiate into vascular endothelial cells, secrete neurotrophic factors, and support neurogenesis (Rizvanov et al., 2008). A recent preclinical trial reported that hUCBMC therapy improved neuromuscular transmission (Souayah et al., 2012). Multiple intravenous injections of hUCBMCs protected MNs and increased the ALS mouse lifespan even when administered in the symptomatic stage, and each cell dose was less than the optimal dose (Garbuzova-Davis et al., 2012). The first case of intramedullary thoracic spinal cord implantation of hUCBMCs in an ALS patient has demonstrated the feasibility of allogeneic transplantation. The study has revealed that the patient does not show persistent structural alterations of the spinal cord during the 3-year follow-up (Cordes et al., 2011). In short, studies have reported the efficacy of hUCBMC therapy in ALS, and it should be further enhanced by genetic engineering in preclinical studies; the next step of conducting clinical trials of hUCBMC therapy is highly important.

As for PBMCs, only a few trials have examined their safety and feasibility for the treatment of ALS. Because PBMCs can release growth factors and regulate the host immune system, they are suitable candidates for cellular therapy in ALS. A small pilot study demonstrated no adverse effects in patients with ALS who are infused with granulocyte-colony stimulating factor–mobilized peripheral blood stem cells (Cashman et al., 2008). Another study that transplanted bone marrow cells and PBMCs separately also confirmed their safety (Martinez et al., 2012). Notably, a recent clinical trial including 14 patients intraspinal transplanted PBMCs into the subarachnoid space and observed no severe transplant-related adverse events (Li et al., 2017). In addition, a case study has shown that no significant clinical changes in ALS patients who transplanted with PBMCs (Janson et al., 2001); allogeneic transplantation of peripheral blood-HSCs in patients with ALS has revealed that cells can enter injured sites of motoneuron in CNS and engraft as immunomodulatory cells, but no clinical benefits (Appel et al., 2008). Based on these safety results, one of the studies provides an aspect that peripheral blood-HSCs could serve as a cellular vehicle for future CNS gene therapy (Appel et al., 2008) and another study suggests that genetically engineered PBMCs could optimize the PBMCs secreting various growth factors to support the injured sites (Janson et al., 2001). Finally, although these studies have been reported the safety of PBMCs, further preclinical studies and larger trials are required to assess their therapeutic efficacy.

### Neural Precursor Cells

The term “neural precursor cell” refers to both neural stem and progenitor cells whose progeny are mature neurons and glial cells (Dibajnia and Morshead 2013). During embryonic development, neuroepithelial cells, which function as NSCs, can be divided into early neurons and radial glial (RG) cells (Gotz and Huttner 2005; Kriegstein and Alvarez-Buylla 2009). RG cells may develop into neurons directly or develop into neurons and glia indirectly through intermediate progenitor cells including GPCs and white matter progenitor cells (Kriegstein and Alvarez-Buylla 2009). Although most of the progenitors can only differentiate into limited phenotypes of glial cells, GPCs can generate both type 1- and type 2-astrocytes and oligodendrocytes (Han et al., 2004; Martins-Macedo et al., 2021). With their multipotent feature, NSCs and GPCs have been frequently discussed as a possible cellular therapy for ALS.

### Neural Stem Cells

Many studies have discussed the therapeutic benefits of NSCs in recent years. Because NSCs can differentiate into neurons, they can replace lost or injured neurons, contributing to the reconstruction of motor circuits (Lepore and Maragakis 2007). NSCs can restore the diseased microenvironment of the CNS through the secretion of neurotrophic factors. Modulation of neuronal plasticity increases the survival and regeneration of endogenous neurons (Kim M. J. et al., 2018), promoting dendritic regrowth (Han et al., 2019), inhibiting apoptotic cell death, and enhancing injured axon regeneration (Keefe et al., 2017). Finally, NSCs release immune-modulatory molecules in the CNS, with a consequential reduction in CD3^+^ T cells and an increase in CD25^+^ and CD25^+^/CD62L^+^ Treg cells (Pluchino and Cossetti 2013). NSCs reduce the proportion of activated macrophages (Pluchino and Cossetti 2013) and interact with mononuclear phagocytes by scavenging extracellular succinate, thus affecting immune responses and suppressing inflammation (Peruzzotti-Jametti et al., 2018).

Preclinical studies have examined the safety and efficacy of NSC implantation. Transplanted rodent NSCs (rNSCs) in SOD1^G93A^ mice showed therapeutic potential through neurogenesis and neurotrophin secretion (Corti et al., 2007). In addition, the efficiency of rNSCs administered through the blood delivery route was higher in symptomatic models (13%) than in presymptomatic models (6%) (Mitrecic et al., 2010). Another study using human NSCs (hNSCs) derived from aborted fetuses reported that hNSCs extensively differentiated into neurons and released GDNF and BDNF, leading to the delay of disease onset and the extension of lifespan (Xu et al., 2006). The combination of FK506 with rapamycin can serve as an effective immunosuppressive regimen (Yan et al., 2006). Grafted hNSCs can not only modulate the motor circuits but also engage in crosstalk with the host ependyma, thus enabling intrinsic repair (Xu et al., 2009; Xu et al., 2012). Moreover, dual transplantations of hNSCs in the cervical and lumbar spinal cord were more effective than single transplantation (Xu et al., 2011), and overexpression of VEGF may significantly improve survival (Hwang et al., 2009). However, the poor therapeutic benefits of transplantation were also reported with neither survival benefit nor delayed disease progression (Hefferan et al., 2012). The reduced survival of engrafted hNSCs may be due to the hostile ALS microenvironment; thus, additional approaches to protect these cells are needed (Srivastava et al., 2017).

To support the translation of NSC therapy from bench to bedside, several clinical trials have been conducted. The first FDA-approved phase I trial was conducted in 2009 (NCT01348451). No major complications were noted after lumbar injections, and most of the adverse events could be attributed to the toxicities of immunosuppressant drugs or the injection procedure itself (Glass et al., 2012); this finding is consistent with that of another phase I study (NCT01640067) (Mazzini et al., 2015). Cervical injections were also well-tolerated, whereas the dual targeting group (both lumbar and cervical) showed slowed disease progression. The transplantation of hNSCs might be beneficial to patients with spinal symptom onset in the early stage of ALS (Tadesse et al., 2014). Autopsies on seven patients showed that transplanted hNSCs could survive up to 2.5 years posttransplant with “nests” consisting of donor cells near the injection site (Glass et al., 2016). Another open-label trial (NCT01730716) indicated that hNSCs could be safely injected at high doses, and the procedure could be expanded to multiple surgical centers (Goutman et al., 2018). Compared with historical controls, transplantation significantly increased the revised ALS Functional Rating Scale scores, which indicated possible efficacy (Mazzini et al., 2015).

Although most studies have reported positive results, several difficulties should be overcome before NSCs become a potent therapeutic tool for ALS. For instance, the poor migration of NSCs is an obstacle. NSCs have a tendency to form cell clusters close to the injection site, which is further enhanced through CXCR4 and adhesion molecules including integrins, selectins, and immunoglobulins (Gonzalez et al., 2016). In addition to the endogenous mechanism, transplantation itself may have latent risks; for example, clots formed by NSCs *in vivo* can cause secondary damage to injected tissues under high-density transplantation (Tang et al., 2017). To summarize, preclinical and clinical studies have provided the framework of engraftment; with more solid knowledge acquired, NSCs may become a preponderant candidate of cellular therapy for ALS.

### Glial Progenitor Cells

GPCs are multipotent cells that can differentiate into oligodendrocytes, as well as type 1 and type 2 astrocytes. Astrocytes and oligodendrocytes have different characteristics, but both play vital roles in ALS pathology. Astrocytes drive MN development, including synaptogenesis and maturation (Allen and Lyons 2018), and they maintain the neuronal microenvironment by mediating ion concentrations, secreting neurotrophic factors, and uptaking neurotransmitters (Liddelow and Barres 2015; Halpern et al., 2019; Izrael et al., 2020). They also provide neuronal protection in ALS models, with preference for the spreading of TDP-43 aggregation from MNs to astrocytes along with better tolerability to aggregates (Smethurst et al., 2020). In addition to protecting MNs from neurotoxicity, astrocytes release exosomes that express neuroprotective proteins with anti-oxidative, anti-apoptotic, and anti-inflammatory effects. With the ability to selectively target neurons, astrocyte-derived exosomes have therapeutic potential by transferring neuroglobin to neurons (Venturini et al., 2019).

Oligodendrocytes play roles in the pathophysiology of ALS (Ferraiuolo et al., 2016). Functioning as myelinating cells, they generate myelin sheaths around axons, thus establishing saltatory conduction and providing both physical protection and metabolic support for neurons (Philips et al., 2013). After myelination, the rate of lactate fermentation increases as a predominant energy source (Funfschilling et al., 2012). Lactate is transported abundantly through monocarboxylate transporter-1 (MCT1), which is highly expressed in oligodendrocytes (Lee et al., 2012), In the disease model of sporadic ALS, MCT1 expression is reduced, causing the death of MNs consequently through two pathways: the deleterious effect on neuronal survival due to MCT1 dysfunction or the homeostatic failure of iron, leading to macrophage cytotoxicity and neuronal ferroptosis (Tognatta and Miller 2016). Because the dysfunction of oligodendrocytes is one of the contributing factors of MN degeneration, the potential of transplanted GPCs to generate oligodendrocytes is now emerging (Walczak et al., 2011).

Targeting therapeutic potential through the differentiation of astrocytes and oligodendrocytes, transplantations of GPCs were conducted in several studies. Engrafted GPCs in the cervical spinal cord of SOD1^G93A^ rats could efficiently differentiate into astrocytes and exert neuroprotection by preventing the loss of GLT1, thus prolonging survival and attenuating the degeneration of forelimb and respiratory MNs (Lepore et al., 2008). However, transplanted human GPCs (hGPCs, also known as Q-Cells) in ALS rodent models did not exert any therapeutic benefits (Lepore et al., 2011). By contrast, several preclinical studies have genetically modified hGPCs to overexpress neurotrophic factors. GDNF-overexpressing hGPCs could survive, integrate, and release GDNF in the spinal cord after transplantation (Klein et al., 2005). The efficient delivery of GDNF significantly enhanced MN survival but had no beneficial effect on functional outcomes (Suzuki et al., 2007), which is consistent with the result of another study (Park et al., 2009). Transplantation of GDNF-overexpressing hGPCs in the SOD1^G93A^ rat motor cortex could delay disease onset and extend lifespan by supporting lower MNs through the releasing GDNF. These cells could also survive and release GDNF in the cortex of cynomolgus macaques (Thomsen et al., 2018). In 2019, a phase I/IIa study (NCT02943850) unilaterally injected hGPCs into the lumbar region of 18 participants. Moreover, a phase I/IIa study (NCT02478450) is currently examining both the safety and efficacy of Q-Cell transplantation in ALS patients and is estimated to be completed in 2024.

Olfactory ensheathing cells (OECs) are a unique class of glial cells because they locate in both the central and peripheral nervous system (Su and He 2010), and could promote axon regeneration in the spinal cord *in vivo* model (Ramón-Cueto and Nieto-Sampedro 1994). Although a preclinical study reveals that OECs intrathecally transplanted into ALS rodent models show no significant benefits than that of in the control rodent (Morita et al., 2008), Li et al. demonstrated that OECs play a neuroprotective function (Li et al., 2011) and prolong survival after the injection of OECs into SOD1^G93A^ rats (Li et al., 2013). As for clinical application, transplantation of OECs has demonstrated that they can slow down the ALS disease progression (Huang et al., 2008). Additionally, a study has shown that 507 ALS patients exhibit partial neurological functional recovery after receiving multiple OECs administration (Chen et al., 2012). However, several authors query the efficacy of OECs treatment because no clear evidences of axonal regeneration, neuronal differentiation, and myelination are seen after post-mortem neuropathologic analyses (Giordana et al., 2010). A prospective study has found no clinical benefits for patients as well (Piepers and van den Berg 2010), which is in line with another observational study showing negative or conflicting outcomes (Gamez et al., 2010). Besides, the safety is still in doubt due to the reported disabling side-effects (Chew et al., 2007). The conflicting results suggested that OECs transplantation should not yet be considered as an alternative treatment for ALS.

## Conclusion and Perspectives

**FIGURE 3:**
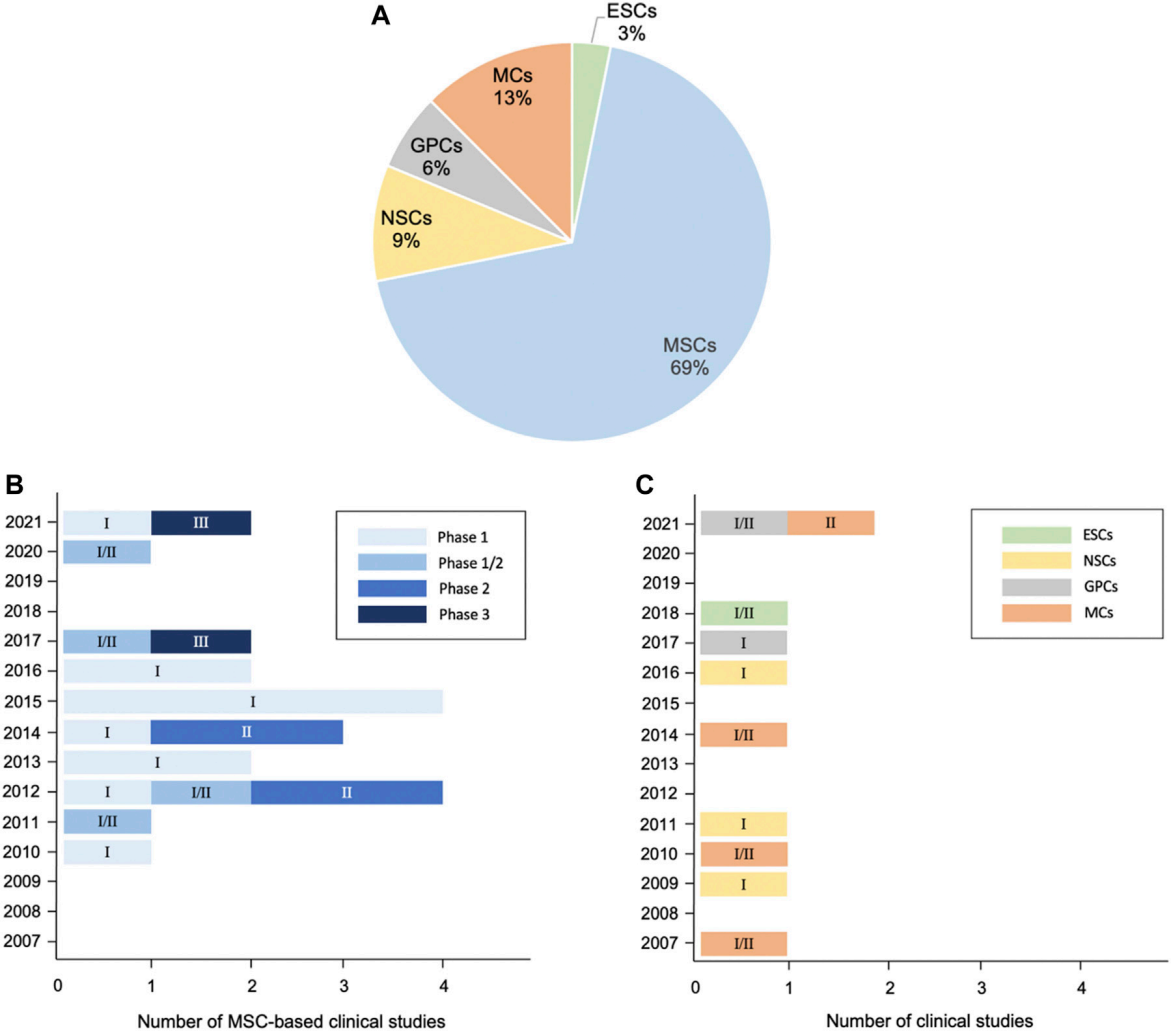
Distribution of cellular therapy for ALS in clinical trials from 2007 to 2021. **(A)** Pie chart: analysis of cell types. **(B)** Trends of MSC-based applications regarding clinical phase. **(C)** Trends of ESC-, neural precursor cell-, and MC-based applications regarding clinical phase. 32 clinical trials have been registered as of October 2021, excluding studies with suspended, temporarily not available, terminated, withdrawn, available, and no longer available status according to clinicaltrials.gov. Numbers in the bars of Figures 3B,C indicate the corresponding phase of clinical trials.

In this review, we summarize potential cellular therapies for ALS, including the pluripotent stem cells ESCs and iPSCs; multipotent stem cells such as MSCs and neuron-related progenitors, as well as MCs. Although ESCs/iPSCs could differentiate into three germ layers, tumor formation risk is the major concern in clinical practice. By contrast, MSCs, neuron-related progenitors, and MCs are promising because of their capabilities of microenvironment modulation and neuroprotection through rich trophic factor secretion, enhanced neurogenesis, abnormal protein aggregate clearance, and immune modulation (Figure 2). The current preclinical studies and clinical trials of cellular therapy on ALS are summarized (Tables 1–3). Among them, MSCs account for the majority of all cell types (Figure 3A). MSCs hold great potential since two phase III studies were conducted in 2017 and 2021 (Figure 3B). Similar to MSCs, mononuclear cells are emerging cellular therapy with one phase II study preparing, whereas neural precursor cells require further clinical research (Figure 3C). Generally, the abundant emergence of clinical trials in recent years reveals the growing interest in the actual application of cellular therapy for ALS. Although most trials remain in the early stages, more advanced studies will be performed, and the cellular therapy platform will optimize the dose and types of different stem cells for future clinical treatment for ALS.
