# Corrigendum: Potential of cellular therapy for ALS: current strategies and future prospects

**DOI:** 10.3389/fcell.2023.1292681

**Published:** 2023-09-19

**Authors:** Ting-Jung Lin, Kuang-Chao Cheng, Luo-Yun Wu, Wei-Yu Lai, Thai-Yen Ling, Yung-Che Kuo, Yen-Hua Huang

**Affiliations:** ^1^ School of Medicine, College of Medicine, Taipei Medical University, Taipei, Taiwan; ^2^ Department of Biochemistry and Molecular Cell Biology, School of Medicine, College of Medicine, Taipei Medical University, Taipei, Taiwan; ^3^ TMU Research Center for Cell Therapy and Regeneration Medicine, Taipei Medical University, Taipei, Taiwan; ^4^ Department and Graduate Institute of Pharmacology, College of Medicine, National Taiwan University, Taipei, Taiwan; ^5^ Research Center for Developmental Biology and Regenerative Medicine, National Taiwan University, Taipei, Taiwan; ^6^ International Ph.D. Program for Cell Therapy and Regeneration Medicine, College of Medicine, Taipei Medical University, Taipei, Taiwan; ^7^ Graduate Institute of Medical Sciences, College of Medicine, Taipei Medical University, Taipei, Taiwan; ^8^ Center for Reproductive Medicine, Taipei Medical University Hospital, Taipei Medical University, Taipei, Taiwan; ^9^ TMU Research Center of Cancer Translational Medicine, Taipei Medical University, Taipei, Taiwan; ^10^ Comprehensive Cancer Center of Taipei Medical University, Taipei, Taiwan; ^11^ PhD Program for Translational Medicine, College of Medical Science and Technology, Taipei Medical University, Taipei, Taiwan

**Keywords:** ALS, mesenchymal stem cell, motor neuron degeneration, precision medicine, cellular therapy

In the published article, an **author** name was incorrectly written as “Guang-Chao Cheng”. The correct spelling is “Kuang-Chao Cheng”. This has been corrected and thereby the citation and author initials in the author contribution section have been corrected.

The authors apologize for this error and state that this does not change the scientific conclusions of the article in any way. The original article has been updated.

